# Aged Microplastics and Antibiotic Resistance Genes: A Review of Aging Effects on Their Interactions

**DOI:** 10.3390/antibiotics13100941

**Published:** 2024-10-06

**Authors:** Kuok Ho Daniel Tang, Ronghua Li

**Affiliations:** 1 Department of Environmental Science, College of Agriculture, Life & Environmental Sciences, The University of Arizona (UA), Tucson, AZ 85721, USA; 2 School of Natural Resources and Environment, UA Microcampus, Northwest A&F University (NWAFU), Yangling 712100, China; rh.lee@nwsuaf.edu.cn; 3 Department of Environmental Science and Engineering, College of Natural Resources and Environment, Northwest A&F University (NWAFU), Yangling 712100, China

**Keywords:** aging, antibiotic resistance genes, biofilm, mechanical fragmentation, microbial community, microplastics

## Abstract

**Background:** Microplastic aging affects the dynamics of antibiotic resistance genes (ARGs) on microplastics, yet no review presents the effects of microplastic aging on the associated ARGs. **Objectives:** This review, therefore, aims to discuss the effects of different types of microplastic aging, as well as the other pollutants on or around microplastics and the chemicals leached from microplastics, on the associated ARGs. **Results:** It highlights that microplastic photoaging generally results in higher sorption of antibiotics and ARGs due to increased microplastic surface area and functional group changes. Photoaging produces reactive oxygen species, facilitating ARG transfer by increasing bacterial cell membrane permeability. Reactive oxygen species can interact with biofilms, suggesting combined effects of microplastic aging on ARGs. The effects of mechanical aging were deduced from studies showing larger microplastics anchoring more ARGs due to rough surfaces. Smaller microplastics from aging penetrate deeper and smaller places and transport ARGs to these places. High temperatures are likely to reduce biofilm mass and ARGs, but the variation of ARGs on microplastics subjected to thermal aging remains unknown due to limited studies. Biotic aging results in biofilm formation on microplastics, and biofilms, often with unique microbial structures, invariably enrich ARGs. Higher oxidative stress promotes ARG transfer in the biofilms due to higher cell membrane permeability. Other environmental pollutants, particularly heavy metals, antibacterial, chlorination by-products, and other functional genes, could increase microplastic-associated ARGs, as do microplastic additives like phthalates and bisphenols. **Conclusions:** This review provides insights into the environmental fate of co-existing microplastics and ARGs under the influences of aging. Further studies could examine the effects of mechanical and thermal MP aging on their interactions with ARGs.

## 1. Introduction

Microplastics (MPs) are tiny plastic particles ranging from 100 nm to 5 mm in size. They are frequently found in the environment due to the large-scale production and improper disposal of plastics [1]. These MPs can absorb and transport common chemical contaminants such as antibiotics and heavy metals, as well as carry microbes like algae and bacteria [2,3]. This complicates their environmental impact and raises concerns about their potential effects on ecosystems and organisms. Upon entering the environment, MPs are exposed to various physical and chemical processes that cause them to age [4]. MPs are synthetic polymers known for their high resistance in the environment. It can be broken down through processes comprising biodegradation, mechanical fragmentation, photoaging, and thermal aging [5]. However, the degradation rate varies significantly depending on the type of MPs, with biodegradable MPs disintegrating much faster than those that are hard to degrade [6]. Aging, in this context, refers to the changes of MPs in the environment over a long period, which primarily involves alterations in surface characteristics, such as crack development, surface morphological changes, and tensile strength reduction. Degradation reflects changes within the microplastic (MP) molecular structure, primarily indicating weight reduction and mass loss of MPs [7].

MPs undergo mechanical fragmentation when external forces cause them to break down into smaller particles [8]. An example of this is the release of MPs from tire wear during car travel on roads [9]. In aquatic environments, MPs could be mechanically fragmented through interactions with sediments and pebbles and the actions of waves and tides. The aging of MPs can also be caused by free radicals and accelerated by exposure to UV radiation in the presence of oxygen. This process, known as photoaging, is the most critical factor contributing to the aging of MPs in the environment [10]. Thermal aging and photoaging differ in terms of the initiators and aging products involved [11]. While photoaging is limited to the exposed surface of MPs, thermal aging can affect the entire MP mass. Furthermore, the initiating chemical reactions vary between the two processes. Thermal aging leads to chain degradation initiated by heat [12]. Biodegradation of MPs occurs through microbial actions, like digestion, and biochemical processes by microorganisms such as bacteria, fungi, and microalgae [8]. These aging processes change the surface characteristics of MPs, facilitating the adsorption of other chemicals in the surroundings and the leaching of additives and polymers from MPs [13,14]. Biodegradation usually starts with the formation of biofilm on the surface of MPs, which then creates a microenvironment conducive to MP biodegradation. MP-associated biofilm, sometimes called the plastisphere, refers to the ecosystem of microbial life that forms on and around plastic debris in aquatic and terrestrial environments [15]. Microorganisms in the biofilm secrete extracellular enzymes that break down polymer chains [4]. However, these processes rarely result in the complete transformation or degradation of MPs, due to the persistent nature of certain MPs [15]. Therefore, aging, rather than degradation, is more commonly used to refer to these processes that subject MPs to physical and chemical changes on their surfaces.

Antibiotic resistance genes (ARGs) are found extensively in both natural and engineered environments [16]. These genes can potentially transfer to pathogenic bacteria through processes like conjugation, transformation, or transduction, posing a significant risk to public health. The wide presence of MPs and ARGs in the environment significantly increases the chances for both contaminants to coexist and interact. In fact, studies have demonstrated that MPs in organisms and environmental media could extend the half-life of antibiotics and promote the formation of ARGs [17,18]. The accumulation of antibiotics and antibiotic-resistant bacteria on MPs has been observed in various environments. MPs can act as a medium for ARGs to transfer between the environment and animals. The formation of biofilms on MPs and their adsorption of pollutants may promote the transfer and evolution of ARGs [19]. Multiple reviews on the interactions of MPs and ARGs have been published over the years. Dong et al. examined the impact of MP and ARG interactions on aquaculture environments [20]. Their review did not include the effects of MP aging on ARGs in the plastisphere. Syranidou and Kalogerakis reviewed the interactions and key factors that influence the sorption process of antibiotics on MPs, particularly in the context of wastewater treatment, rather than how MP aging affects the sorption of ARGs. [21]. Zhang et al. were more interested in how MPs in the mariculture system enhance ARGs [18]. Yu et al. undertook a meta-analysis of ARG abundance on MPs in aquatic settings [22], aiming to quantitatively clarify the enrichment effect and compare it to non-MP materials. Zheng et al. delved into the intricate mechanisms through which MPs entwined with biofilm contribute to the proliferation of ARGs [23]. Furthermore, the review of Liu et al. focuses on the roles of MPs in enriching and transporting ARGs [24]. They did not explain the changes in the interactions between MPs and ARGs as MPs age. Additionally, they examine the processes that specifically facilitate the accumulation of a greater number of ARGs. The extant reviews invariably focus on the occurrence of ARGs in different locations and environmental media and the effects of MP and ARG interactions. There is no review on how the aging of MPs, particularly the adsorption of other chemicals and the leaching of chemicals from MPs, affects the ARGs in the plastisphere.

This review, therefore, aims to examine the effects of MP aging, including photoaging, mechanical fragmentation, thermal aging, and biotic aging, on ARGs. It also reviews the effects of environmental pollutants prone to sorption by MPs and chemicals leached from MPs on ARGs in the plastisphere, as well as the mechanisms of interactions involved. Since MPs undergo continuous changes in the environment due to aging, this review could provide a better understanding of the dynamics of ARGs on aging MPs and how these affect the spreading of ARGs and the associated risks.

## 2. Photoaging of MPs and the Effects on ARGs

**Figure 1:**
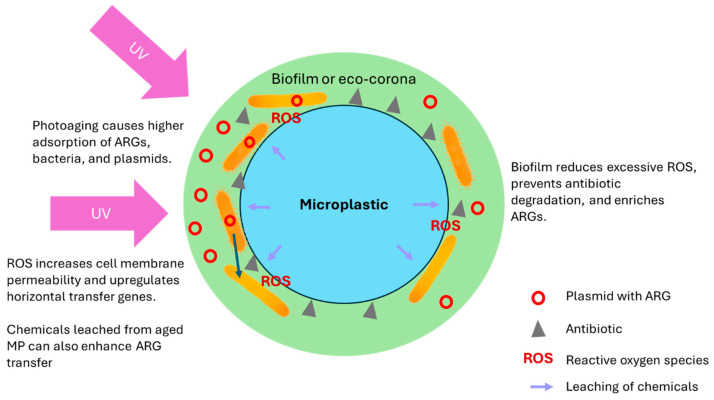
The effects of MP photoaging on ARGs on the MP surface.

The consequences of MP aging on the fate of ARGs in the plastisphere have not been subjected to thorough research. In a recent study, Yuan et al. used UV-aged polystyrene (PS)-MPs to investigate the impact of aging on the transfer of ARGs to bacteria [25]. They found that the adsorption capacity of aged MPs for *Escherichia coli*, plasmids, and phage lambda-carrying ARGs increased significantly, leading to enhanced ARG transfer frequency (Figure 1). The increase was likely because of the higher specific surface area and ARG affinity of the MPs after photoaging [25]. Additionally, the release of organic compounds during aging induced intracellular reactive oxygen species generation and upregulated genes associated with horizontal gene transfer, resulting in a substantial increase in the ARG transfer from the carriers, particularly the plasmid (Figure 1). The enhanced ARG transfer likely resulted from the synergy of higher bacterial adsorption on and chemical leaching from aged MPs (Figure 1) [25]. A study revealed that the ability of tetracycline to be adsorbed onto MPs was substantially increased by the photoaging process [26]. At 288 K, the adsorption capacity at equilibrium rose from 0.387 to 0.507 mg/g, and at 308 K, it increased from 0.507 to 0.688 mg/g. The improved adsorption capacity was due to the acquisition of more high-energy adsorption sites as MPs photoaged. Moreover, the enhanced adsorption of tetracycline promoted the development of ARGs in the biofilm associated with the MPs, namely *tetA*, *tetB*, *tetC*, *tetD*, *tetE*, *tetG*, and *tetK* [26].

Similarly, Guo et al. observed that exposure of MPs to UV light increased their ability to adsorb antibiotic resistance plasmids by 42.7–48.0% [27]. This exposure resulted in a shift from monolayer to multilayer adsorption. The authors attributed these effects to the increase in surface roughness and the presence of oxygen-containing functional groups on the MPs after UV exposure [27]. Furthermore, they found that the eco-corona of MPs in wastewater subjected to UV treatment contained 1.33–1.55 times more ARGs than in wastewater not treated with UV (Figure 1). The plasmids might have adsorbed onto the MPs via electrostatic physisorption. As MPs photoaged, the polarity of their oxygen-containing groups weakens, thus facilitating the electrostatic attraction of negatively charged plasmids. Higher pH and lower salinity promoted the desorption of the plasmids, causing higher ARG abundance in the eco-corona [27]. The eco-corona on MPs is the term used to describe the first layer of biomolecular compounds that attach to the surface after being exposed to the environment [28]. The eco-corona changes the surface characteristics of MPs, which can markedly impact how they group together, move, spread in living organisms, form biofilms, and produce harmful effects [29]. The UV-aging of PS in a different study also led to significant changes in the PS MPs, characterized by increased carbonyl index, hydrophilicity, hydroxyl radical content, and specific surface area. Notably, the UV-aged PS had an adsorption capacity for erythromycin that was almost 8 times higher than the original PS [30]. Compared to the original PS, the biofilms on the UV-aged PS had a higher cell count (5.6 × 10^8^ CFU/mg vs. 1.4 × 10^8^ CFU/mg), with resistance mutation occurring more frequently (1.0 × 10^−7^ vs. 1.4 × 10^−8^). Additionally, when erythromycin was present at 0.1–1.0 mg/L, resistance mutation in the biofilms on the original and UV-aged PS was significantly enhanced [30].

While photoaging tends to increase the abundance of ARGs in the biofilm associated with MPs, hence their spread in the environment, a contrary observation was made on PS nanoparticles. After exposure to simulated sunlight, numerous enzyme-like activities, possibly involving peroxidase and oxidase, were detected on photo-aged PS nanoparticles, causing the release of reactive oxygen species. At 0.1 and 1 μg/mL, original and aged PS nanoparticles aid plasmid (pUC19 and pHSG396) transfer within *E. coli* because of a moderate amount of reactive oxygen species generated, which increased the permeability of cell membrane (Figure 1) [31]. When the aged PS nanoparticle level increased to 5 and 10 μg/mL, plasmid transformation was hindered due to excessive reactive oxygen species, particularly hydroxyl and superoxide radicals produced by nanozyme activities. This could disrupt plasmid structure and degrade ARGs. The harmful free radicals also caused oxidative damage to the bacteria, disintegrating their cell membranes, reducing their SOS response, and limiting the production of adenosine triphosphate. This ultimately led to the inactivation of the affected cells [31]. The photoaging of MPs was reported to alter the plastisphere by lowering the hydrophobicity of MPs. Upon photoaging, the bacterial community structure in the plastisphere was observed to become less complex and stable, while its carbon metabolic capacity increased [32]. The dominant bacterial genus in the MPs changed from *Achromobacter* to *Burkholderia*. These changes may have implications for the ARGs. *Burkholderia cepacia*, for instance, is a known Gram-negative pathogen occupying the plastisphere, which can be resistant to multiple antibiotics [33]. However, there is very little research relating the changes in the biofilm bacterial communities to the dynamics of ARGs.

Ding et al. investigated how original tire wear particles and biofilm-anchoring tire wear particles affect the photodegradation of tetracycline when exposed to simulated sunlight [34]. They observed that both types of tire wear particles enhanced the photodegradation of tetracycline, with the original particles showing a higher rate (0.0232 h^−1^) of photodegradation compared to biofilm-anchoring particles (0.0152 h^−1^). The increased efficiency of tetracycline photodegradation was attributed to the differing levels of reactive oxygen species produced by the two types of tire wear particles when exposed to sunlight [34]. Specifically, original particles generated more reactive oxygen species, including hydroxyl radicals and superoxide anions, which are crucial for the breakdown of tetracycline. The original particles were photosensitized to a greater extent compared to the biofilm-anchoring particles [34]. Though this study is not related to the effects of photoaging on ARGs, it confirms the role of reactive oxygen species, particularly the hydroxyl and superoxide radicals, in degrading antibiotics, and demonstrates that biofilm could reduce reactive oxygen species on MPs exposed to sunlight (Figure 1). This may explain why the plastisphere could facilitate the accumulation of antibiotics, giving rise to selective pressure on the microbial communities therein and facilitating the propagation of ARGs.

The presence of other MPs could also affect the photoaging of a particular MP and the microbial community structure of MP biofilm. A study found that the dissolved organic matter from two biodegradable plastics, namely polybutylene adipate terephthalate and polycaprolactone, can quicken the breakdown and oxidation of PS MPs exposed to light in the same medium. The dissolved organic matter of polybutylene terephthalate is protein-like, while that of polycaprolactone contains tryptophan and tyrosine components, resulting in different photochemical activities, with the former promoting the generation of singlet oxygen and superoxide radicals [35]. These reactive oxygen species could cause faster degradation of the PS backbone. The combined effect of PS photoaging and dissolved organic matter from biodegradable plastics notably enhanced the diversity, metabolism, and richness of the microbial community [35]. This study indicates that photoaging could be beneficial for biofilm development. However, it does not explain if the enhanced biofilm development promoted ARG propagation and if the reactive oxygen species from co-existing biodegradable plastics affected the ARG abundance. Klumper et al. conducted a study on the correlation between microbial diversity and antibiotic resistance and found higher microbial diversity, evenness, and richness in soil demonstrated a significant negative correlation with the relative abundance of ARGs. The enhanced microbial community could decrease the persistence of ARGs in soil [36].

A recent study examined how different levels of aging in PS MPs affected the breakdown of sulfamethoxazole in water under simulated sunlight. The results demonstrated that the PS MPs hindered the natural breakdown of sulfamethoxazole when exposed to sunlight, with the rate of breakdown decreasing as the aging level of PS MPs increased [37]. It was found that aged PS MPs caused a reduction in the breakdown of sulfamethoxazole by creating a light-screening effect. Further experiments showed that this hindrance in the breakdown of sulfamethoxazole was due to a decrease in the triplet-excited state of sulfamethoxazole. Aged PS MPs were also found to form more unsaturated chromophores and generate organic intermediate compounds, leading to increased absorption of photons [37]. This aligns with the findings of Ding et al., which state that original MPs caused higher photodegradation of tetracycline than aged biofilm-anchoring MPs because their higher photosensitivity led to a higher generation of reactive oxygen species [34]. This implies that the photoaging of MPs could potentially increase the abundance and propagation of ARGs by slowing down the photodegradation of antibiotics and increasing the adsorption capacity of MPs for ARGs. In fact, an adsorption study confirmed that, as MPs age, they become more effective at adsorbing pollutants, specifically levofloxacin [38]. The isothermal adsorption process can be well represented by both the Langmuir and Freundlich models, suggesting that it involves both single- and multiple-molecular-layer adsorption. Experimental results indicate that the adsorption process is influenced by mechanisms such as electrostatic interaction, hydrogen bonding, π-π conjugation, and ion exchange [38].

In summary, it can be concluded that MP photoaging results in higher adsorption of antibiotics and ARGs. Aged MPs demonstrate higher adsorption for ARGs or agents carrying ARGs due to the increase in specific surface area and the alteration of functional groups on the surface of MPs that facilitate their interactions with plastids and ARGs. Additionally, the ability of aged MPs to adsorb antibiotics better and slow down the photodegradation of antibiotics results in the development of ARGs within the biofilm. However, nanoplastics may exhibit exceptional behaviors, as their exposure to sunlight can induce the release of excessive reactive oxygen species, which can damage plasmids and ARGs. At low concentrations, reactive oxygen species promote ARG transfer through upregulating genes associated with horizontal gene transfer and increasing cell membrane permeability. Photoaging is also likely to affect the biofilm microbial community structure and the dynamics of ARGs in the biofilm, though the results have been inconclusive, partly due to environmental factors. Nonetheless, the presence of biofilm on MPs could slow down the photodegradation of antibiotics and facilitate the formation of ARGs.

## 3. Mechanical and Thermal Aging of MPs and the Effects on ARGs

**Figure 2:**
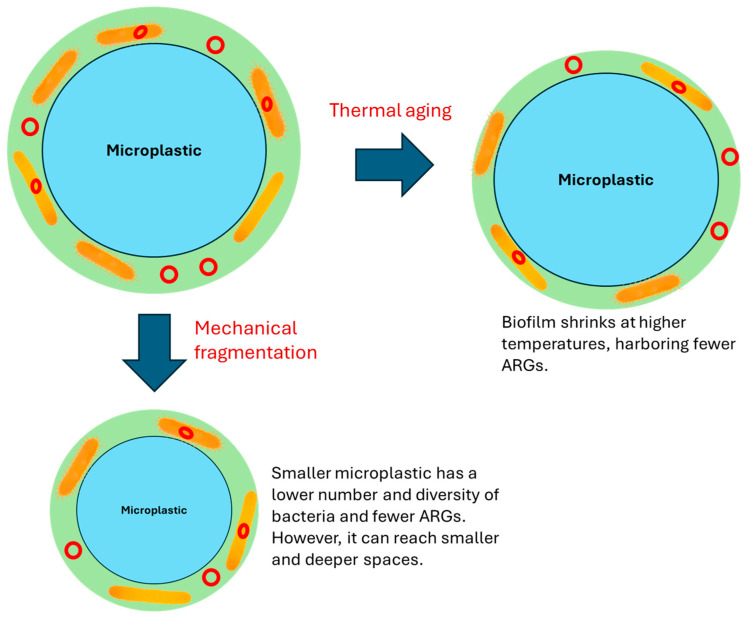
The effects of mechanical fragmentation and thermal aging on MP-associated ARGs.

Currently, studies on how mechanical and thermal aging affects the dynamics of ARGs on MPs are limited. Mechanical aging mainly results in the size reduction of MPs and is therefore related to how the size of MPs influences the ARGs thereon [39]. Wang et al. reported that exposing wastewater continuously to MPs did not have a negative impact on the removal of nutrients, but it did change the occurrence patterns of ARGs [40]. Polyamide and polyethylene terephthalate MPs in the mm size range caused an increase in ARG quantities by 42.8% and 54.3%, while those in the μm size range led to an increase of 31.3% and 39.4%, respectively. This indicates that mm-MPs had a more pronounced effect on ARGs compared to μm-MPs [40]. After prolonged exposure, the surface properties of mm-MPs were significantly altered, which facilitated the attachment of microbes. Furthermore, the sludge with mm-MPs showed a greater number of taxonomic connections compared to sludge with μm-MPs, with potentially different host bacteria for ARGs (Figure 2) [40]. The rough surface of MPs and the close relationships between ARGs and bacterial taxa promoted the spread of ARGs, explaining the higher abundances observed in mm-MPs [40,41]. Mechanical fragmentation of MPs may produce more μm-MPs with reduced ARG abundance.

The aging of MPs in soil results in their downward transport and fragmentation. Yan et al. researched how polypropylene MPs found in farmland soil aged and moved downward in natural loamy sand [42]. They revealed significant aging of the MPs, producing tiny plastic fibers and carbonyl groups. The MPs were also found to have mixed with soil minerals. In their tests, they observed that the MPs mixed with humic acid traveled the farthest downward, followed by the raw MP with soil minerals and, finally, the clean MP without soil minerals [42]. They also found that the surface charge and density of the MPs were important factors affecting their movement. At the maximum distance the MPs traveled, the authors only found smaller rod-shaped plastic fibers, indicating that the aging process had caused the plastic to break down into smaller pieces. Additionally, they detected three classes of ARGs (beta-lactam, sulfonamide, and tetracycline) on the surface of the MPs, suggesting that the MPs could potentially transport these genes to deeper layers of the soil [42]. Therefore, the mechanical aging of MPs could transport ARGs deeper into the soil, although it is uncertain how the ARG abundance changes when the MPs fragment on their way down the soil (Figure 2).

Similarly, no studies have been conducted to investigate the effects of MP thermal aging on the associated ARGs. However, studies have related biofilm on MPs to a greater abundance of ARGs [43]. The role of MP biofilm in harboring and enriching ARGs has been well established. The direct absorption of antibiotic residues and ARGs by MP biofilm is a crucial factor in enriching ARGs [44]. Absorption of antibiotic residues by MP biofilm results in higher levels of antibiotic residues within the biofilm [45,46]. MP biofilm provides the medium for the interaction between antibiotics and microorganisms, leading to the selective enrichment of antibiotic-resistant bacteria and ARGs in MP biofilms when high levels of antibiotics are present in the surroundings [47]. The abundance of ARGs in biofilm is, therefore, closely linked to the microbial communities in MP biofilm and the type and concentrations of antibiotics. Understanding how the biofilm changes with temperature variation could indirectly shed light on the effects of thermal MP aging on ARGs. A study found that biofilm amounts on plastics (polyethylene, polylactic acid, and polypropylene pellets) varied by season and location, influenced mainly by seawater temperature [48]. Different biological communities, especially diatoms and bacteria, were observed on the plastic surfaces. Microbial communities varied with seasons and locations, with bacteria on MPs exhibiting distinct metabolisms. Biomass of the biofilm was observed to decrease with temperature along with enzymatic reactions and cell metabolism (Figure 2) [48]. Temperature seems to have a greater influence than dissolved oxygen and nutrients on the seasonal biomass change in the MP biofilm [49]. This suggests that MPs exposed to heat may experience a decrease in the biomass of the associated biofilm, which may have an inhibitory effect on ARG propagation since ARG abundance is closely linked to biofilm (Figure 2).

Along the same vein, a study probing the effect of changing temperature on ARGs during anaerobic digestion indicated that during the process of thermophilic digestion (55 °C), 8 out of 10 identified ARGs decreased [50]. In contrast, only four ARGs decreased during moderate digestion (20 °C) and five during mesophilic digestion (35 °C). The changes in ARGs and bacterial communities were similar in the moderate and mesophilic treatments but differed from those in the thermophilic system. The succession of bacterial communities during anaerobic digestion was identified as the primary mechanism influencing the changes in ARGs and integrons [50]. However, studies on the effect of higher temperatures on the abundance of ARGs do not always yield consistent results. For instance, Lin et al. observed a negative correlation between temperature and the relative abundance of ARGs in swine manure, which was not obvious in chicken manure, whereas Huang et al. found that high temperatures did not consistently reduce ARGs during the anaerobic digestion of swine manure [51,52]. This indicates that the interrelationship between MPs, the plastisphere, the ambient temperature, and ARGs is complex. Furthermore, little is known about the ARG dynamics on MPs subjected to thermal aging.

Generally, limited studies exist that directly examine the effects of MP mechanical and thermal aging on the dynamics of ARGs. Studies conducted in other related settings often need to be referred to extract the relevant information. Larger MPs in sludge were found to have more abundant ARGs, probably because of the rough surface and the close relationships between ARGs and bacterial taxa. This implies that the reduction in the size of MPs as they age could lead to reduced ARG abundance and the transport of ARG to deeper layers of soil and sediment since smaller MPs can move further down these media. Higher temperatures could negatively influence the MP biofilm by reducing its biomass or altering the succession of bacterial communities, resulting in a reduction in ARG abundance. However, composting at higher temperatures did not consistently produce lower ARG abundance. It remains largely unknown how the abundance and distribution of ARGs change on MPs that are undergoing thermal aging or already thermally aged. These gaps constitute a prospective research area.

## 4. Biotic Aging of MPs and the Effects on ARGs

Biotic aging of MPs mainly centers on the growth of biofilms on MPs that change their surface morphology and characteristics. The MP biofilm is sometimes called the plastisphere, meaning the microbial communities developing and dwelling on plastic materials, including MPs [4]. While microbial communities colonize MPs, they may be able to use the carbon source provided by the plastic polymer, causing the biodegradation of MPs. The biodegradation rates differ among different polymer types, with hard-to-degrade plastics often experiencing very limited degradation [15]. This area is perhaps the most widely studied as far as the effects of MP aging on ARGs are concerned. MP biofilm could selectively enrich ARGs. Wu et al. examined microbial communities and antibiotic resistance genes in biofilms on different substrates, namely MPs, rocks, and leaves [53]. They found that the biofilm on MPs had a unique structure and a distinct range of ARGs and pathogens, such as *Pseudomonas monteilii* and *Pseudomonas mendocina*, not found in biofilms on natural substrates. This implies that MP biofilm may function as a carrier of ARGs and pathogens. In parallel to this, Liu et al. also observed that when the biofilms of polyethylene MPs and quartz sand were subjected to the selective pressures of sulfamethoxazole, tetracycline, and zinc at rising concentrations, the ARGs in the former were more significantly enhanced than the latter, resulting in distinct bacterial communities between the biofilms of both materials [54]. In a similar study examining biofilm development and structure on MPs and natural surfaces, it was found that biofilms significantly grew over time on various substrates, with MPs exhibiting a higher biofilm formation than stone. When assessing antibiotic resistance, *tetB* was found to be selectively enriched on polypropylene and polyethylene terephthalate MPs [55]. A comparison of the bacterial communities between the MPs, natural wood particles, sediment, and water of the Ganjiang River, China, revealed that the bacterial communities on MPs were significantly more diverse and richer than those in water but were similar to wood particles. Bacteria of the genera *Flavobacterium*, *Janthinobacterium*, *Pseudomona*, and *Rhodoferax* were more abundant on MPs than in water and sediment. MPs offer a unique microbial habitat with distinctive compositional and functional profiles from natural wood, sediment, and water. The human pathogenic bacteria on MPs also differed significantly from those in water and sediment, characterized by the domination of the *Pseudomonas* genus [56].

MP biofilms have been observed to accelerate the transformation of extracellular ARGs naturally (up to 1000 times higher frequency) in the presence of single or multiple bacterial species when compared to the biofilms on natural substrates [53]. Small and aged MPs were also found to have significantly higher ARG transformation frequencies than large and original MPs. Moreover, the occurrence of transformation events within MP biofilms demonstrated a strong positive correlation with both bacterial density and the content of extracellular polymeric substances [53]. While Wang et al. reported that larger MPs could adsorb more microbes and ARGs, this study demonstrated that the transformation of ARGs is faster in small and aged MPs [40,53].

The selective pressure of antibiotics in the environment could affect ARGs in MP biofilm. When ciprofloxacin is present, microorganisms in both the water and MP biofilm are more likely to carry ARGs at higher levels (by 2–3 times), which contributes to the enrichment of ARGs [57]. The initial formation of MP biofilm might inhibit the transfer and proliferation of ARGs compared to the surrounding water. However, with the pressure exerted by ciprofloxacin, the MP biofilm tends to concentrate ARGs more than the surrounding water, acting as a buffer sphere that stabilizes ARG levels [57]. In fact, the antibiotic-resistant bacteria found in MP biofilms could be 100 to 5000 times more abundant than in the surrounding environment [18]. Microbes inside MP biofilms are more densely packed and in closer physical contact [58]. The presence of a greater number of secretory system genes and increased cell motility within MP biofilms lead to frequent interactions between cells [59]. Furthermore, the frequency of plasmid transfer within MP biofilms can be significantly higher than among the free microorganisms in the surroundings [58]. Studies have also observed a strong positive relationship between the presence of the Class 1 integron gene and the abundance of ARGs in MP biofilms [18,60]. In addition, the development of MP biofilms boosts the presence of hydrophilic components like C–O and C=O on the MP surface, which aids in attaching outsider cells and ARGs, as well as the uptake of organic substances through polar interactions [61]. The sturdy biofilm structure also offers physical support and safeguards against external mechanical harm during the movement of MPs. This increases the resilience of the agents hosting ARGs in MP biofilms to stress, thereby presenting a greater risk of persistence [23].

The role of MP biofilms in enriching ARGs during anaerobic sludge digestion has also been reported. Different forms of MPs were found to cause an increase in the proportion of ARGs in an aerobic sludge digester. As the number of MPs increased from 10 to 80 particles per gram of total solids (g-TS), the ARG level rose from 4.5 to 27.9% compared to the control [62]. The ARG levels in the sludge digester were influenced by the structure and type of MP polymer. Specifically, antibiotic-resistant bacteria and ARGs were enriched in the biofilm associated with low-density polyethylene (LDPE). The biofilm provided a conducive environment for the growth of antibiotic-resistant bacteria and the enhancement of functional genes, thus facilitating the vertical transfer of ARGs. Horizontal transfer was also enhanced when LDPE was present at 80 particles/g-TS, as indicated by higher levels of mobile genetic elements and plasmids with ARGs [62]. Higher oxidative stress, cell cohesion, and cell membrane permeability also aided the horizontal transfer of ARGs [62]. This is parallel to the study of Chen et al., which found that moderate oxidative stress is beneficial for plasmid transfer due to increased cell membrane permeability [31]. Furthermore, Feng et al. observed the development of biofilms on four different types of MPs [63]. They found ARGs on these biofilms, thus confirming the role of MP biofilms in anchoring ARGs. These MPs were placed in both aerobic and anaerobic tanks at a wastewater treatment plant for incubation. The study revealed that the various types of MPs showed different abilities in enriching bacteria and ARGs compared to the surrounding environment. The researchers also observed a higher frequency of horizontal ARG transfer in MP biofilms in the water samples taken from both tanks, confirming that MPs could facilitate horizontal ARG transfer [63].

Likewise, a separate study revealed that *Aeromonas*, *Bacillus*, and *Pseudomonas* were the most prevalent bacterial genera among antibiotic-resistant bacteria in the treated wastewater [64]. The analysis of viable antibiotic-resistant bacteria showed their abundance increased in the MP biofilm during incubation with PS beads from day 3 to day 5, followed by a decrease on days 7 and 9. The presence of antibiotic-resistant bacteria in the MP biofilm varied depending on duration and the total organic carbon content while generally showing minimal effects from a slight temperature variation, low antibiotic pressure, and higher MP mass [64]. Significant variations in the relative abundance of MP biofilm-associated ARGs such as *vanA* and *sul1* and integron integrase gene (intl1) were observed when the antibiotic pressure, time, and total organic carbon content were varied [64]. The aging of MPs in wastewater treatment processes has invariably resulted in the increased abundance of the associated ARGs. In wastewater treatment, using a constructed wetland, the variety of microorganisms on MPs increased as time passed [65]. Initially, the composition of biofilms from sewage changed more in the wastewater effluent than in the constructed wetland. Following treatment involving both conventional and constructed wetland methods, the presence of pathogens and antibiotic resistance decreased significantly [65]. However, when MP material inoculated with sewage was introduced to the constructed wetland, the impact was less pronounced. *Aeromonas*, *Klebsiella*, and *Streptococcus* were identified as important pathogenic genera associated with antibiotic resistance in the biofilms on MPs. Although there was a decrease in human pathogens and antibiotic resistance during the treatment process, biofilms on MPs remained a significant source of antibiotic resistance (*intI1* gene) [65].

The biotic aging of MPs in natural environments has also been observed to enhance ARGs. A study conducted in coral reef ecosystems revealed that the variety of bacteria found on MP surfaces was lower when compared to those in the surrounding seawater, and the types of bacteria in the two environments were different [66]. Upon further investigation, it was discovered that some bacteria on the MPs carried genes that make them resistant to antibiotics, and the prevalence of these genes was associated with the concentration of antibiotics in the seawater [66]. For instance, the presence of *Vibrio* bacteria was found to be linked to the presence of *sul1* genes on MP biofilms in areas with higher levels of sulfonamides. These findings imply that MPs may preferentially attract and support the growth of bacteria from reef environments, potentially leading to the increased abundance of ARGs [66]. The same was observed in urban rivers. Despite having lower bacterial diversity than the surroundings, the bacterial community composition on MPs was substantially different from the surrounding water and ARGs were enriched on MPs. The integron-integrase genes were found to be more prevalent in MPs, possibly indicating a heightened propensity for horizontal gene transfer. This suggests that MPs function as a unique habitat for microorganisms and may promote microbial interactions [47].

In short, there is relatively more information about the effects of the biotic aging of MPs through biofilm formation on ARGs than other types of MP aging. MP biofilms have mostly been linked to the enrichment and higher abundance of ARGs, harboring unique microbial structures different from their surroundings. MP biofilms usually demonstrate higher transformation and transfer of ARGs than biofilms on the natural substrates and the surroundings. In some circumstances, the higher transfer could be facilitated by higher oxidative stress that increases cell membrane permeability, as with MP photoaging. The presence of other substances or pollutants in the surroundings could affect the ARGs in MP biofilms, where total organic carbon content and antibiotics, especially the latter, were found to increase the abundance of ARGs in MP biofilms. This is discussed further in the subsequent section on how chemicals from MPs and their surroundings affect ARGs. Enrichment of ARGs in MP biofilms was observed in both natural and non-natural environments.

## 5. Effects of Environmental Chemicals and Leaching on ARGs

MP aging is characterized by the leaching of plastic additives and polymer degradation products from MPs and the adsorption of environmental chemicals by MPs [67]. While it is generally known that antibiotics in the environment could promote the formation of ARGs in MP biofilms, this section aims to provide a broader examination of the effects of different pollutants and chemicals on MP-associated ARGs. Liu et al. used a combination of sulfamethoxazole, tetracycline, and zinc to observe their effects on the development of ARGs, mobile resistance genes, and bacterial communities in biofilms [54]. The presence of these substances, particularly metal pressure, led to a significantly higher increase in ARG abundance in the biofilms associated with MPs than those of natural substrates (Table 1) [54]. The study revealed clear differences in the bacterial communities of the two biofilm types, as well as in the core bacterial species and the occurrence of ARGs. Furthermore, the research indicates that MP biofilms potentially contain more bacterial hosts of ARGs, and metal pressure plays a role in facilitating the spread of ARGs (*int1*) through co-selection (Table 1) [54].

In aquaculture environments, the presence of both antibiotics and heavy metals can lead to bacteria developing resistance to antibiotics through co-selection, cross-resistance, and other mechanisms [79]. Additionally, MPs can serve as hotspots for the enrichment and transmission of antibiotic resistance in aquaculture settings. This can result in multidrug-resistant microorganisms infiltrating aquatic organisms through the food chain and ultimately posing a significant risk to both aquaculture and human health [80]. An investigation into the effects of ampicillin and copper in sewage on polyethylene MPs and polyethylene MPs contaminated with triclocarban revealed that the presence of triclocarban on polyethylene MPs at a level of 2.48 mg/g did not hinder bacterial adhesion and biofilm formation [68]. In fact, it could promote the adhesion of certain pathogens, particularly *Aquabacterium* and *Pseudoxanthomonas*, better than uncontaminated MPs. The presence of triclocarban also led to elevated levels of multiple ARGs in both biofilms and sewage. The combined presence of triclocarban, ampicillin, and copper appeared to exert a stronger selective pressure on bacterial communities, promoting the co-selection of resistance genes. Additionally, tetracycline-contaminated polyethylene MPs led to increased mobile genetic elements in sewage (*IS613*, *trb-C*, *tnpA-04*, *intl1*, and *intl3*), potentially facilitating the propagation and transfer of ARGs (Table 1) [68].

The presence of surfactant in the environment might also affect ARG transmission via MPs. Sun et al. utilized sophorolipid, a type of surfactant, to accelerate the degradation of tetracycline and *tet* genes alongside MPs in soil within a greenhouse environment [69]. Over a period of 49 days, they observed that soil bacteria and phages played crucial roles as reservoirs for ARGs. Additionally, they noted that the presence of MPs significantly hindered the degradation of tetracycline and ARGs in the soil. However, the application of sophorolipid was found to counteract this negative impact of MPs, resulting in the greatest degradation of tetracycline and ARGs in the soil (Table 1) [69]. This implies that, unlike heavy metals, antibacterial agents, and antibiotics, surfactants might have a positive effect on reducing ARGs by aiding in the dispersion and degradation of antibiotics. Nonetheless, more studies are needed to confirm the effects of surfactants on MP-associated ARGs.

A recent study focused on soil has shown that the bacterial community present on tire particles differed significantly from the surrounding soils. This bacterial community exhibited lower diversity and was notably influenced by exposure to heavy metals and antibiotics (Table 1) [70]. This is consistent with other studies that the bacterial diversity in MP biofilms is usually lower than in their surroundings, and the community structures in both environments are distinct [54,66,71]. The study also identified a varied collection of ARGs on tire particles, and their prevalence notably increased under the combined stress of heavy metal and antibiotic exposure, indicating a substantial combined effect. Additionally, the study established a clear connection between the bacterial community and the presence of ARGs on tire particles. It indicates that exposure to heavy metals and antibiotics could potentially escalate the prevalence of ARGs on tire particles, posing a risk to soil ecosystems and human health [70]. When struvite-loaded zeolite was introduced as a soil amendment material, it significantly decreased the bioavailability of copper and tetracycline by at least 73.0% and 71.3%, respectively, in soil contaminated with 1% MPs (Table 1) [72]. Additionally, it resulted in a 76.2–80.3% decrease in the overall relative abundance of ARGs. The presence of struvite-loaded zeolite correlated with a significant decrease in the relative abundance of certain ARGs, namely *intl1*, *sul1*, *tetG*, and *tetX*, which were linked to the availability of copper and tetracycline in the soil [72]. The lower accumulation of pollutants due to struvite-loaded zeolite reduced the selection pressure for MP-associated ARGs. Struvite-loaded zeolite indirectly mitigated the proliferation of ARGs in soil polluted by MPs because it influences soil properties, soil microbial structures, and pollutant bioavailability [72].

In another study focusing on the effects of landfill leachate treatment processes, particularly chlorination and Fenton oxidation, on the removal and regrowth of ARGs on MPs and in leachate, the levels of ARGs on MPs saw a reduction of 34.0–46.3% after chlorination and 92.1–97.3% after Fenton oxidation, in comparison to the respective 54.3–77.6% and >99.9% reduction in the leachate (Table 1) [73]. Following chlorination, there was a notable regrowth of ARGs in the leachate after 48 h. ARG regrowth on MPs was observed to be up to 17 times higher than the leachate. Contrastingly, Fenton oxidation led to a lower regrowth of target ARGs. The results indicate that it is harder to eliminate ARGs from MPs than leachate. Both leachate and especially MPs have the ability to quickly regrow ARGs after being treated with chlorine [73]. The presence of chlorine residues in water will likely spur the regrowth of ARGs, especially those associated with MP biofilms. Not only is the abundance of MP-associated ARGs affected (in most cases enhanced) by environmental pollutants, but it is also affected by other functional genes. In a sequencing batch reactor, the presence of PS MPs at 0.5 to 50 mg/L resulted in increased diversity and richness of the microbial community therein, along with a significant enhancement of the denitrification process [74]. Furthermore, at lower concentrations (0.5–5 mg/L), PS MPs were observed to stimulate the secretion of extracellular polymeric substances and elevate the expression of functional genes linked to carbon fixation, carbon degradation, nitrogen cycling, and phosphorus cycling (Table 1) [74]. Notably, the presence of PS MPs at lower levels led to a 27.13% increase in *aac(3)-II*, a 38.36% increase in *blaTEM-1*, and a 9.57% increase in *tetW*. The total absolute abundance of *intI1* increased nearly twofold. Intriguingly, 78.4% of the functional genes related to C, N, and P displayed a positive correlation with *intI1*, which suggests a favorable environment for the transmission of ARGs. This study sheds light on the intricate connections between the functional components of wastewater treatment plants and the propagation of ARGs under the influence of MPs [74].

Studies that specifically examine the effects of chemicals leached from MPs on the ARGs in the plastisphere are non-existent. There are studies that investigate how plastic additives influence ARG dynamics, but they are limited. In an anaerobic sludge digester deliberately contaminated with different concentrations of dimethyl phthalate, the impact of 0.1 mg/L of dimethyl phthalate on methane production was observed to be minimal, whereas 10.0 mg/L of dimethyl phthalate enhanced methanogenesis by aiding in the breakdown of sludge (Table 1) [75]. At the higher concentration, dimethyl phthalate encouraged the growth of methanogens and maintained the prevalence of the multidrug resistance gene in the ARGs profile. Furthermore, it elevated the levels of several predominant mobile genetic elements, hinting at dimethyl phthalate’s potential to amplify the spread of ARGs. In contrast, the lower concentration of dimethyl phthalate (0.1 mg/L) did not alter the levels of ARGs and mobile genetic elements. Finally, the combined exposure of dimethyl phthalate and antibiotic stress showed that 10.0 mg/L of dimethyl phthalate actively contributed to the propagation of ARGs (Table 1) [75]. In a drinking water supply setting, when polypropylene plastic pipes were exposed to different conditions, such as chlorination, heating, and freeze–thawing, faster formation of MPs and chemical leaching occurred [76]. The MPs came in various shapes and aggregation states, while the profiles of chemical leachates were distinctive because of different physicochemical treatments. The exposure to leachates destabilized the microbial community and increased the dominance of pathogenic bacteria (Table 1) [76]. The properties of leachates, such as pollutant content, MPs, organic carbon, and zeta potential, significantly influenced genes related to antibiotic resistance and virulence, strengthening the interaction between ARGs, pathogenic bacteria, and virulence genes [76]. While this study did not aim to examine the alteration in ARG dynamics due to chemical leaching resulting from the natural aging of MPs, it provides highly relevant information related to the effects of chemicals leached from plastic pipes subjected to different water treatment processes on plastic-associated ARGs.

Bisphenols are popular plasticizers. At their environmentally relevant concentrations, four bisphenols (bisphenol A, S, F, and AF) can substantially boost the proliferation of antibiotic ARGs in *Acinetobacter baylyi* ADP1 by 2.97–3.56 times (Table 1) [77]. The uptake of resistance plasmids and metabolic adjustment in ADP1 contributed primarily to their development of resistance. These metabolic changes involved increasing the production of capsule polysaccharides and intracellular metabolic enzymes, resulting in thicker capsules that could trap free plasmids better [77]. At the same time, genes responsible for DNA uptake and translocation were upregulated so that plastids with antibiotic resistance could undergo natural transformation better [77]. Wu et al. were also interested in the effects of dimethyl phthalate on ARGs. They found that at environmentally relevant concentrations ranging from 2 to 50 μg/L, dimethyl phthalate was observed to significantly enhance the transfer of ARGs among bacteria present in intrageneric (3.82 times), intergeneric (4.96 times), and wastewater (4.77 times) microbiota (Table 1) [78]. The study findings indicate that dimethyl phthalate reacts strongly with the cell membrane’s phosphatidylcholine bilayer, resulting in reduced membrane lipid fluidity and higher membrane permeability, which facilitate ARG transfer. Furthermore, the stress caused by dimethyl phthalate also led to an increase in the production of reactive oxygen species and overexpression of genes associated with conjugative gene transfer, contributing to the observed increase in gene transfer [78].

It becomes increasingly clear that environmental pollutants can alter the dynamics of MP-associated ARGs, in addition to the selective pressure already exerted by antibiotics. The effects of heavy metals, on top of antibiotics, have been most widely studied, and they synergistically lead to the increased abundance of ARGs in MP biofilms, probably via co-selection and cross-resistance. While most studies are conducted in aqueous environments, studies on soil also produce similar results, highlighting the enhanced prevalence of ARGs on MPs. The presence of surfactants and adsorbents might counteract the effects of MPs by mitigating ARG proliferation in water and soil. These materials could be present in the environment, but they are often added for remediation. Chlorination from water treatment could give rise to residues and boost ARGs in MP biofilms. Other co-existing functional genes may have a similar effect. Very few studies directly investigate how chemicals leached from MPs change the associated ARGs. Studies using the additives themselves and leachates from plastic pipes generally show a positive effect on ARG proliferation and transfer. Therefore, the content in this section is in parallel with that of the previous sections that the aging of MPs most likely increases ARG prevalence in the associated biofilm and, specifically, the adsorption of pollutants from the surroundings and the leaching of chemicals from aging MPs also produce the same outcome.

## 6. Discussion

Currently, the reviews on ARGs associated with MPs have invariably focused on their environmental occurrence, fate, interactions, and impacts. The reviews on how MP aging alters the dynamics of ARGs on MPs are extremely limited. Yet, it is crucial to put the relevant information together systematically to permit a better understanding of how MP aging affects the associated ARGs. This is because MPs typically undergo aging in the environment, typified by photoaging, mechanical fragmentation, thermal degradation, and biofilm formation. MP aging alters MP properties, the exposure of MPs to environmental pollutants, and chemical leaching from MPs, influencing ARG dynamics on MPs.

Photoaging of MPs increases the adsorption of antibiotics and ARGs, probably due to an increase in specific surface area, changes in functional groups on the MP surface that may confer MPs more adsorption sites or alter their adsorption behaviors, and the leaching of organic compounds from MPs, which facilitates horizontal gene transfer [23,26,27]. The improved ability of aged MPs in adsorbing antibiotics correlates positively with the abundance of ARGs selectively induced by the antibiotics adsorbed [30]. However, nanoplastics could have a contrary effect on ARGs by generating excessive reactive oxygen species at higher concentrations [31], thus destroying ARGs, whose transfer is generally facilitated at lower concentrations of reactive oxygen species capable of increasing cell membrane permeability. This implies the size-dependent effect of plastic particles on ARG abundance, which is linked to reactive oxygen species level. Reactive oxygen species from MP photoaging could also directly degrade antibiotics, alleviating the selective pressure for ARG proliferation [34]. The photosensitivity of MPs has been highlighted as a crucial factor in determining the amount of reactive oxygen species generated [35,37]. More studies are required to confirm these complex effects. Similarly, photoaging is likely to destabilize or alter microbial community structure in the biofilm and the dynamics of ARGs [32,33], but the mechanisms have yet to be elucidated.

There is a lack of direct evidence demonstrating the effects of mechanical and thermal aging on MP-ARG interactions. Larger MPs (typically of mm size range) in sludge have more abundant ARGs, possibly due to their rough surface and close relationships with the corresponding bacterial taxa that promote ARG spread [40,41]. Smaller MPs may transport ARGs to deeper layers of soil and sediment. This is evident during MP aging in soil, resulting in the downward transport of progressively fragmented or aged MPs. Rod-shaped plastic microfibers were reported to travel the most down the soil, and they may facilitate the transfer of ARGs to deeper soil layers [42]. Higher temperatures were found to shrink MP-associated biofilms and reduce ARG abundance, likely due to reduced bacterial growth, compromised extracellular polymeric substances at high temperatures affecting biofilm stability, decreased horizontal gene transfer, and the degradation of ARGs [48,49]. However, composting studies yield inconsistent results, showing that higher temperatures did not consistently produce lower ARG abundance [50,51,52]. They are largely constrained by the limited temperatures examined and the substrates used, which might not represent MPs well.

The biotic aging of MPs through biofilm formation on ARGs has been studied more than other forms of aging. MP biofilms show higher transformation and transfer of ARGs compared to biofilms on natural substrates [53,54,55]. MP biofilms, particularly those of small and aged MPs, could accelerate the transformation of extracellular ARGs naturally. The presence of other substances or pollutants in the surroundings could also affect the ARGs in MP biofilms. Environmental antibiotics have been reported to exert selective pressure that facilitates ARG proliferation [57]. Enrichment of ARGs in MP biofilms has been observed in both natural and non-natural environments. Biotic aging of MPs may counteract the reactive oxygen species produced by photoaging, slowing down the breakdown of antibiotics and ARGs. On the contrary, MP photoaging could alter the biofilm structure [32,33,34]. The combined effects of different types of MP aging require further verification.

Environmental pollutants can influence the ARGs associated with MPs, in addition to the selective pressure exerted by antibiotics and heavy metals [54,68,70]. Studies show that environmental pollutants can increase the abundance of ARG in MP biofilms (Table 1), although surfactants and adsorbents may counteract these effects [69,72]. Chlorination during water treatment and chemicals leached from MPs also enhance ARG proliferation [73]. This further elucidates the complex effects of different types of MP aging on ARGs, with biotic aging potentially reducing the reactive oxygen species from photoaging and photoaging altering biofilm dynamics. Biotic aging could also enhance the sorption of various chemicals with synergistic or antagonistic effects on ARG proliferation.

This review has identified multiple gaps that need to be filled in so that the effects of MP aging on the associated ARGs can be better comprehended. Firstly, it would be necessary to experimentally examine how mechanical fragmentation of MPs affects the ARG dynamics or the relationship between different MP sizes and ARG abundance on MPs. Secondly, the changes in ARGs on MPs in response to their thermal degradation require further investigation. This could involve exposing MPs to different environmentally relevant temperatures to observe how the associated biofilms and ARGs change. Thirdly, it may be vital to study how various environmental pollutants that MPs adsorb can interact with and affect the ARGs on MPs. Additionally, since MPs contain numerous additives, the effects of different plastic additives on ARGs could be examined. Further study could explore the relationship between chemicals leached from aging MPs and the ARGs on MPs. It is also intriguing to untangle the complex nexus between photoaging, biotic aging, MP photosensitivity, and reactive oxygen species, as well as their collective effects on biofilm structure, antibiotics on MPs, and ARGs.

## 7. Conclusions

Upon entering the environment, MPs are subjected to aging processes, potentially altering their physicochemical and biological properties, hence their interactions with ARGs. MP photoaging frequently results in higher adsorption of antibiotics and ARGs and the production of reactive oxygen species at moderate levels that could facilitate horizontal gene transfer via increasing cell membrane permeability. Larger MPs could harbor more ARGs, and smaller MPs resulting from aging facilitate the transport of ARGs deeper into soil and sediments. Indirect evidence on the effect of temperature on ARGs is inconsistent, where higher temperatures do not always lead to lower ARG abundance. Biotic aging of MPs is perhaps the most widely studied, and it is generally agreed that MP biofilms enrich ARGs. Environmental pollutants comprising heavy metals, antibiotics, chlorination residues, and other functional genes could potentially enhance MP-associated ARGs, as do chemicals leached from MPs, such as bisphenols and phthalates. Surfactants and adsorbents, often used as remediation agents, produce the opposite effects. This review contributes to a better understanding of how surface changes of aged microplastics alter their ability to carry ARGs and the structure of biofilms on their surfaces, which also affects their potential for proliferating ARGs. It sheds light on the environmental fate of MPs and ARGs, which have been shown to co-exist due to their prevalence. The focus on MP aging provides a more realistic picture of how environmental MPs interact with ARGs under the influence of aging.

## Figures and Tables

**Table 1:** The effects of different environmental chemicals and plastic additives on ARGs.

MP Type	Chemical	Effect	Reference
Polyethylene (3–4 mm diameter)	Sulfamethoxazole, tetracycline, zinc	Increased selective colonization of ARG host bacteria was observed; more abundant ARGs were present in MP biofilms; the spread of *intl1* was enhanced.	[54]
Polyethylene (116 µm in diameter)	Contaminated with triclocarban and placed in sewage containing ampicillin and copper	More abundant pathogenic bacteria were found on MPs contaminated with triclocarban; the MPs also led to more ARGs in biofilm and sewage.	[68]
MPs from greenhouse films	Sophorolipid and tetracycline	MPs increased the persistence of tetracycline and ARGs in soil; sophorolipid dissipated soil tetracycline and ARGs and reduced their soil levels.	[69]
Tire particles (13–1400 µm)	Copper and tetracycline	Diverse ARGs were present on tire particles; their abundance increased when the particles were exposed to copper and tetracycline.	[70]
Polyethylene	Chloramphenicol, sulfamerazine, tetracycline, tylosin	ARGs were significantly enriched on MPs; MP-associated ARGs and antibiotics decreased as the salinity of waterbodies increased.	[71]
Environmental MPs	Struvite-loaded zeolite, copper, tetracycline	Struvite-loaded zeolite lowered the bioavailability of copper (>73%) and tetracycline in soil (>71.3%); it reduced the levels of ARGs (up to 80.3%) in soil; higher levels of bioavailable copper and tetracycline led to higher levels of most ARGs.	[72]
Polystyrene (1 mm)	Chlorination and Fenton oxidation	ARGs on MPs were more persistent and were reduced by up to 46.3% after chlorination, in comparison to up to 77.6% for free ARGs; Fenton oxidation reduced up to 46.3% MP-bound ARGs. ARGs on MPs regrew significantly (up to 17 times) after chlorination.	[73]
Polystyrene (80–160 µm)	P-, N-, and C-related functional genes in a sequencing batch reactor	MPs increased the abundance and diversity of microorganisms in the reactor; at 0.5–5 mg/L, MPs raised the expression of P-, N-, and C-related functional genes, and *aac(3)-II* (27.1%), *bla_TEM_-1* (38.4%), and *tet*W (9.6%); there was a positive correlation between the functional genes and ARGs.	[74]
None; this study examined the effects of a plastic additive on ARGs	Dimethyl phthalate in a sludge anaerobic digester, antibiotics (sulfadimidine, erythromycin, or tetracycline)	10 mg/L dimethyl phthalate raised the levels of certain predominant mobile genetic elements; together with antibiotics, it increased ARG propagation.	[75]
Polypropylene from pipe	Leachate from plastic pipes subjected to chlorination, heating, and freeze–thawing	All leachate samples led to a higher abundance and prevalence of pathogenic bacteria; pipe leachate from chlorination positively correlated with ARGs, pathogenic bacteria, and virulence genes.	[76]
None; this study examined the effects of plastic additives on ARGs	Bisphenol A, S, F, and AF	Bisphenols increased ARG transfer in *Acinetobacter baylyi* by up to 3.56 times through incorporating plasmids, alteration of cellular metabolisms, and the actions of reactive oxygen species.	[77]
None; this study examined the effects of a plastic additive on ARGs	Dimethyl phthalate	2–50 µg/L of dimethyl phthalate increased ARG transmission among intrageneric (3.82 times), intergeneric (4.96 times), and wastewater (4.77 times) microbiota through plasmid-mediated conjugation; dimethyl phthalate reduced the fluidity of membrane lipid and caused the membrane to be more permeable.	[78]

## Data Availability

No new data were created or analyzed in this study. Data sharing is not applicable to this article.
